# Temperature-Dependent Evolutionary Speed Shapes the Evolution of Biodiversity Patterns Across Tetrapod Radiations

**DOI:** 10.1093/sysbio/syac048

**Published:** 2022-07-09

**Authors:** A Skeels, W Bach, O Hagen, W Jetz, L Pellissier

**Affiliations:** Department of Environmental Systems Sciences, Landscape Ecology, Institute of Terrestrial Ecosystems, ETH Zürich, Zurich 8092, Switzerland; Swiss Federal Institute for Forest, Snow and Landscape Research WSL, Birmensdorf 8903, Switzerland; Department of Environmental Systems Sciences, Landscape Ecology, Institute of Terrestrial Ecosystems, ETH Zürich, Zurich 8092, Switzerland; Swiss Federal Institute for Forest, Snow and Landscape Research WSL, Birmensdorf 8903, Switzerland; Department of Environmental Systems Sciences, Landscape Ecology, Institute of Terrestrial Ecosystems, ETH Zürich, Zurich 8092, Switzerland; Swiss Federal Institute for Forest, Snow and Landscape Research WSL, Birmensdorf 8903, Switzerland; German Centre for Integrative Biodiversity Research (iDiv) Halle-Jena-Leipzig, Leipzig 04103, Germany; Department of Ecology and Evolutionary Biology, Yale University, New Haven, CT 06520, USA; Center for Biodiversity and Global Change, Yale University, New Haven, CT 06520, USA; Department of Environmental Systems Sciences, Landscape Ecology, Institute of Terrestrial Ecosystems, ETH Zürich, Zurich 8092, Switzerland; Swiss Federal Institute for Forest, Snow and Landscape Research WSL, Birmensdorf 8903, Switzerland

## Abstract

Biodiversity varies predictably with environmental energy around the globe, but the underlaying mechanisms remain incompletely understood. The evolutionary speed hypothesis predicts that environmental kinetic energy shapes variation in speciation rates through temperature- or life history-dependent rates of evolution. To test whether variation in evolutionary speed can explain the relationship between energy and biodiversity in birds, mammals, amphibians, and reptiles, we simulated diversification over 65 myr of geological and climatic change with a spatially explicit eco-evolutionary simulation model. We modeled four distinct evolutionary scenarios in which speciation-completion rates were dependent on temperature (M1), life history (M2), temperature and life history (M3), or were independent of temperature and life-history (M0). To assess the agreement between simulated and empirical data, we performed model selection by fitting supervised machine learning models to multidimensional biodiversity patterns. We show that a model with temperature-dependent rates of speciation (M1) consistently had the strongest support. In contrast to statistical inferences, which showed no general relationships between temperature and speciation rates in tetrapods, we demonstrate how process-based modeling can disentangle the causes behind empirical biodiversity patterns. Our study highlights how environmental energy has played a fundamental role in the evolution of biodiversity over deep time. [Biogeography; diversification; machine learning; macroevolution; molecular evolution; simulation.]

Environmental energy is a fundamental requirement for the growth and persistence of organisms and often a positive predictor of terrestrial biodiversity at broad spatial scales (Currie 1991; Waide et al. 1999; Allen et al. 2012). As such, the relationship between energy and organismal biology, including metabolism and growth rate, has been proposed as a key feature of a unified theory of biodiversity (Brown 2004; Stegen et al. 2009; Brown et al. 2014). However, the way in which environmental energy shapes the evolution of biodiversity is still debated. The evolutionary speed hypothesis (ESH) (Rensch 1959; Rohde 1992) proposes that different rates of biological processes, including molecular and phenotypic evolution, result in different speciation rates between environments with high- and low kinetic energy (temperature; Allen et al. 2007), such as between tropical and temperate biomes, leading to an uneven distribution of biodiversity across the globe. There are multiple possible pathways predicted by the ESH (Fig. 1): a direct temperature dependency of the origin of biological variation through mutation (M1); a direct life-history dependency of the rate in which variation passes between generations through time, or in other words the nucleotide generation time (Martin and Palumbi 1993) (M2); and an interacting effect of temperature and life history, such that rates are highest in lineages with faster life histories occupying warmer areas (M3). Faster rates in one of these pathways could accelerate the formation of reproductive barriers or ecological differentiation in diverging populations, leading to speciation. It is expected that multiple biodiversity patterns, including the distribution of speciation rates across phylogenies or throughout geographic space, will emerge from variation in evolutionary speed through space and time (Fig. 1), and this could additionally be influenced by major fluctuations in environmental energy and temperature over the deep time scales (Condamine et al. 2013; Meseguer and Condamine 2020). Therefore, a holistic integration of patterns and processes in the context of a dynamic Earth history may provide mechanistic support for the role of environmental energy in the evolution of life on Earth and the establishment of biodiversity patterns.

**Figure 1.**
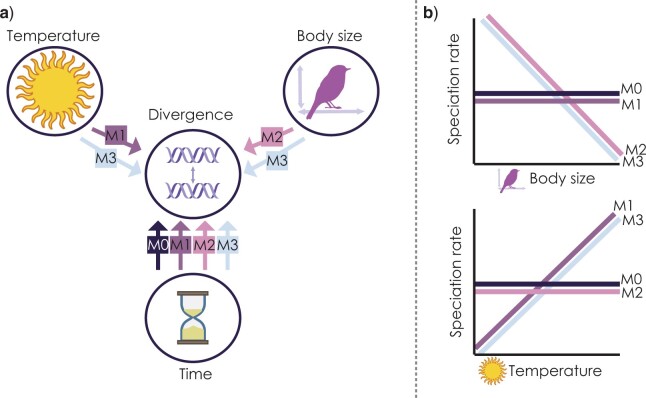
The evolutionary speed hypothesis (ESH) predicts alternative pathways for variation in the rate of population divergence and speciation, driven by temperature (as a measure of environmental kinetic energy) or body size (as a proxy for life history speed). a) Alternative models of population divergence under the ESH: M0—population divergence is a function of time from initial divergence; M1—population divergence is a function of time and the average temperature across each population’s geographic range; M2—population divergence is a function of time and the average body size of each population (as a proxy for life history); M3—population divergence is a function of time, temperature, and body size. b) Predictions for variation in speciation rates with respect to temperature and body size based on alternative models of the ESH.

The kinetic energy of the environment could shape evolutionary speed via different pathways, but it has been difficult to find consistent support for the microevolutionary processes underpinning the ESH. Environmental energy can increase mutation rates from degenerative effects of UV radiation (reviewed in Hua and Bromham 2017) or from increased oxidation from free radicals associated with elevated metabolic rates (Martin and Palumbi 1993; Gillooly et al. 2005; Gillooly and Allen 2007). If mutations are fixed within a population, then energy will drive the rate at which populations diverge and phenotypes change in the absence of gene flow. The nature of the relationship between energy (or proxies thereof) and rates of molecular evolution is mixed. A positive relationship is supported across a broad range of taxa, including fish (Wright et al. 2011), amphibians (Wright et al. 2010), birds (Gillman et al. 2012), and mammals (Gillman et al. 2009), lizards (Ivan et al. 2022), turtles (Lourenço et al. 2013), angiosperms (Davies et al. 2004; Bromham et al. 2015), and foraminifera (Allen et al. 2006). However, these relationships are not universal (Bromham and Cardillo 2003; Orton et al. 2019) and in some cases may be explained by covariation between temperature and life-history traits such as longevity (Hua et al. 2015). The underlying mechanism, such as metabolic rates, also show inconsistent relationships with rates of molecular evolution (Lanfear et al. 2007; Santos 2012). These alternative results could be related to the choice of active or resting metabolic rate as the dependent variable (Gillman and Wright 2013), or may instead be because enhanced DNA repair mechanisms are selected for in harsh environments, compensating for increased mutation rates (Ries et al. 2000; Lynch 2010; Hua et al. 2015; Svetec et al. 2016; Hua and Bromham 2017). Temperature can further drive faster growth rates and shorter generation times, which lead to more cell divisions and recombination events, resulting in a greater turnover of genetic material per unit of time (Rohde 1992; Martin and Palumbi 1993; Dowle et al. 2013). High rates of molecular evolution are associated with fast life-history syndromes (e.g. small body sizes, shorter generation times) in a number of clades (Bromham et al. 1996; Bromham 2002; Qiu et al. 2014; Barrera-Redondo et al. 2018) and with high rates of cell division (Lanfear et al. 2013) (but see [Weir and Schluter 2008a]). If life-history, temperature, or both together can drive rates of molecular evolution, then they may directly influence rates of diversification (reviewed in Dowle et al. 2013; Hua and Bromham 2017).

The ESH links microevolutionary processes controlling the origin and fixation of biological variation to macroevolutionary patterns of lineage diversification and biodiversity gradients throughout geological history. However, the relationship is complex and integrates several interacting ecological and evolutionary processes. Mutations that are fixed in a population can drive speciation based on a Bateson–Dobzhansky–Muller model of divergence in which reproductive isolation results from incompatible genomes in genetically differentiated populations (Dobzhansky 1982; Coyne and Orr 2004). Alternatively, higher mutation rates might increase the standing genetic variation in a population for natural selection to act upon, driving ecological speciation (Schluter and Conte 2009). Therefore, although the specific mechanisms are debated, the core prediction of the ESH is that faster rates of speciation occur in high-temperature environments. Higher diversification rates have been found in several clades occupying lower latitudes and high-temperature environments (Cardillo 1999), but there is also mounting evidence that diversification rates are dynamic in both space and time (Meseguer and Condamine 2020), reflecting major changes in Earth’s climate and geology (Condamine et al. 2013). In some cases, diversification rates are decoupled or even negatively related to environmental energy (Rabosky et al. 2015, 2018; Quintero and Jetz 2018; Economo et al. 2019). There is also evidence that the formation of reproductive isolation is not the limiting factor in the formation of new species on deep time-scales (Rabosky and Matute 2013). This has led to the view that there is currently no unambiguous evidence that the ESH drives biodiversity patterns such as the latitudinal diversity gradient (Rabosky 2021). To gain insight into the factors that shape variation in evolutionary speed, we must consider microevolutionary drivers of diversification in the context of a dynamic Earth history and alongside other potential drivers of speciation. Although this is difficult to achieve with empirical data on present day biodiversity, simulation models that can integrate complementary biological, ecological, and evolutionary mechanisms over deep-time could offer a key tool to contrast alternative pathways and generate predictions regarding the formation of biodiversity gradients (Descombes et al. 2018; Rangel et al. 2018; Pontarp et al. 2019; Saupe et al. 2019; Hagen, Flück et al. 2021).

Here, we assessed how variation in evolutionary speed shapes emergent biodiversity patterns in tetrapods (birds, mammals, amphibians, and reptiles) using a simulation-based inference approach (Fig. 2), which provides a new angle to investigate a challenging hypothesis. We implemented alternative ESH models (Fig. 1) in a spatial, macroevolutionary simulation engine (Hagen, Flück et al. 2021), accounting for temporal and spatial variation in environmental kinetic energy via paleo-reconstructions of temperature over the Cenozoic period (Hagen et al. 2019; Hagen, Flück et al. 2021). The simulations followed the diversification of lineages on a gridded landscape based on paleoenvironmental reconstructions of global temperature, aridity, and plate tectonics (Fig. 2a), where variation in speciation rates emerges from the speed in which populations diverge in allopatry (Fig. 2b). We contrasted four models (Fig. 1) where the rate of population divergence is driven by temperature (M1), body size (as a proxy for life history speed; M2), and both temperature and body size together (M3), as well as a null model in which divergence is unrelated to body size or temperature (M0). We evaluated model support in tetrapods using supervised machine learning tools based on multiple spatial, phylogenetic, and trait-based biodiversity metrics. These alternative models provided a nested framework to test the interacting effect of temperature and life history on divergence, which is important for distinguishing between drivers of variation in evolutionary speed. For example, the metabolic theory of ecology predicts support for a body-size-only model of divergence (M2) for endotherms, whose body temperature is independent of environmental temperature, but support for a temperature and body-size model of divergence (M3) for ectotherms, who regulate body temperature externally. Specifically, we asked: (i) are there general, empirical correlations between environmental temperature, species richness, body size, and speciation rates in tetrapods that might support the ESH? (ii) How do biodiversity metrics differ between simulated ESH models? (iii) Using simulation-based inference, what is the relative support for the ESH in tetrapods based on multidimensional biodiversity patterns?

**Figure 2.**
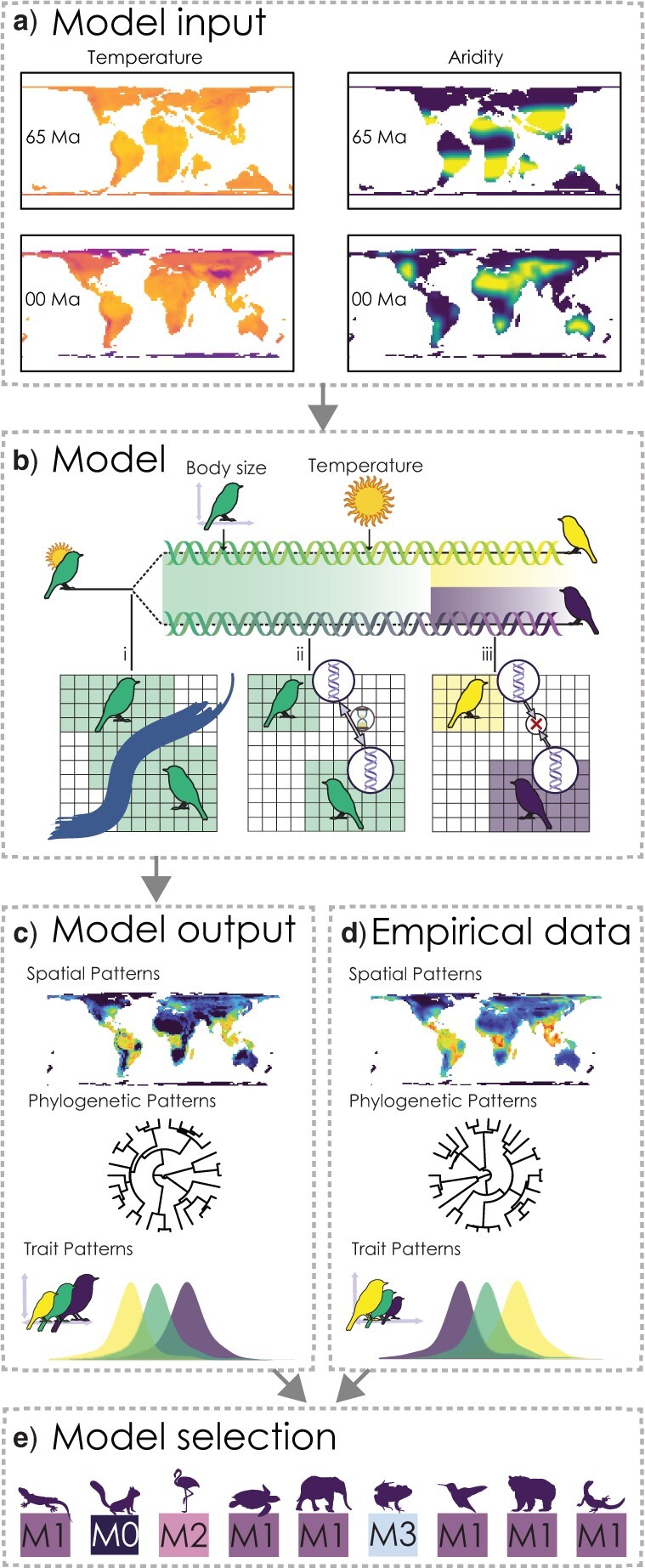
Simulation-based inference framework for testing the evolutionary speed hypothesis (ESH). Diversification is modeled over macroevolutionary timescales on a dynamic gridded landscape using the *gen3sis* simulation engine. a) Paleo-temperature and paleo-aridity reconstructions at $\sim$170 kyr intervals from 65 Ma to the present day are used as the domain for the simulation. b) Speciation begins when populations of a species become geographically isolated (i). These populations diverge from one another through time. The rate of divergence is a function of time (M0), time and temperature (M1), time and body size (M2), or time, temperature, and body size (M3; ii). Diverging populations become distinct species after a threshold of differentiation is reached (iii). c) Biodiversity patterns emerge from the distribution of species, phylogenetic relationships, and the evolution of traits and are summarized using different summary statistics. d) Empirical biodiversity patterns in tetrapods are summarized using the same set of summary statistics as for the simulated data based on species distributions, phylogenetic relationships, and functional traits. e) Supervised machine learning classification algorithms are trained on the simulated summary statistics from four different population divergence models (M0–M3) and are used to perform model selection on the empirical summary statistics.

## Materials and Methods

### Empirical Data and Biodiversity Summary Statistics

We selected independent evolutionary radiations of tetrapods to test the ESH at the level of taxonomic order for birds, mammals, and amphibians, as well as for testudines and crocodilian reptiles. We divided squamate reptiles, the most diverse terrestrial vertebrate order, into six infraorders (hereafter referred to as orders) as this group was significantly more diverse than other orders and a taxonomic classification above the family-level was available for this group (Meiri 2018). We selected taxonomic orders as the unit of comparison because the ages of these clades are mostly closely distributed around 65 Ma allowing for a fair comparison with simulated data (Supplementary Fig. S1) as well as being readily biologically and ecologically interpretable. Some clades, however, are significantly older than 65 Ma (Supplementary Fig. S1). To test whether our results might be biased by the variance in clade age, we dissect the maximum clade credibility (MCC) phylogenetic tree for each taxonomic class at 65 Ma and repeat the model selection procedure (see below) on all subtrees (henceforth referred to as time-slice clades), ensuring clade age was consistent in simulated and empirical data. We found the results to be qualitatively similar for the time-slice clade analyses and we present these in Supplementary Appendix S4, whereas here focusing on the order-level analysis.

We obtained matching data on the geographic distribution and phylogenetic position of extant species of terrestrial vertebrates collected through the VertLife project (vertlife.org) in association with Map of Life (mol.org). Phylogenies were downloaded from VertLife and follow (Jetz et al. 2012; Tonini et al. 2016; Jetz and Pyron 2018; Upham et al. 2019; Colston et al. 2020). Distribution data for birds came from (Jetz et al. 2012) and for reptiles from the Global Assessment of Reptile Distributions (Roll et al. 2017). For mammals and amphibians, we modified distributions from the International Union for Conservation of Nature (International Union for Conservation of Nature 2016) to match the names of the respective phylogenies, and for squamate reptiles, we matched names following (Skeels et al. 2019). We performed this step manually for crocodilia and testudines. We aggregated spatial distributions into 220 km $\times $ 220 km equal-area grid cells to match the spatial resolution of the simulated dataset. We obtained body-size data from a variety of sources for birds (Wilman et al. 2014), mammals (Wilman et al. 2014; Faurby et al. 2018; Cooke et al. 2019; Etard et al. 2020), amphibians (Oliveira et al. 2017; Etard et al. 2020), and reptiles (Meiri 2008, 2010, 2018; Feldman et al. 2016; Colston et al. 2020). This dataset comprised of matching phylogenetic, spatial, and body size data for 32,024 species including, 9991 species of birds, 6677 amphibians, 9859 reptiles, and 5497 mammals. We then selected all orders ($n = 48$), and time-slice clades ($n = 164$) with more than 20 species for further analyses. We use 20 species as a threshold because summary statistics calculated on small samples may show spurious patterns. The retained orders varied in their diversity between 20 and 5966 species (mean $= 604$).

There are many ways to summarize biodiversity patterns, and we selected 54 different summary statistics that capture phylogenetic, spatial, and functional trait-based dimensions of biodiversity and can be assigned as a single numeric value to each clade (Supplementary Table S1). We focused on body size as a phenotypic trait that is ecologically significant and covaries with life history across taxa (Promislow et al. 1992). We also acknowledge that the relationship between life-history traits (such as generation time) and body size is variable across taxa (Promislow et al. 1992); however, it remains one of the best available proxies for a wide range of species across vertebrate taxa. Body size might drive variation in diversification rates independently of a generation time effect (e.g., via metabolic rates [Gillooly et al. 2005]). As such, support for body-size dependent model would be suggestive of an effect of generation time. Additionally, we estimated species’ temperature niches using the mean value of mean annual temperature (CHELSA; Karger et al. 2017) across all 220 km $\times $ 220 km grid cells within a species’ geographic range. The 54 biodiversity summary statistic metrics could be broadly categorized into four classes: (i) species-specific phylogenetic metric correlations, including correlations between mean root distance (MRD) and body size; (ii) spatial metric correlations, including correlations between species richness, temperature, latitude, and phylogenetic and phenotypic diversity across 220 km $\times $ 220 km equal-area grid cells (see the Supplementary Appendix SI for a discussion of spatial scale); (iii) trait metric correlations and distributions, including properties of the frequency distribution of species’ temperature niche or body size; (iv) phylogenetic tree size and shape metrics, including clade size, tree imbalance, and the distribution of node heights. For the order-level analysis, phylogenetic metrics were calculated either as the mean value across 50 trees sampled randomly from the posterior distribution for each class, or as s single value from the MCC tree, before subsequently pruning species without matching spatial or trait data for downstream analysis. We compared the impact of these two methods on model selection (see below). For the time-slice clade analysis, phylogenetic metrics were only sampled on the MCC tree.

The ESH is associated with well-constrained predictions about the distribution of biodiversity in space and variation in diversification rates across lineages (Fig. 1), and before exploring the support for the ESH using the simulation-based approach, we asked to what degree these predictions were supported in tetrapods using a traditional correlative approach. We used meta-analytical tools to test for general patterns in four of these key summary statistics based on Spearman correlations across tetrapod taxa: (i) coefficient of correlation between species richness and latitude across grid-cells; (ii) correlation between species richness and temperature across grid-cells; (iii) correlation between speciation rate and body size across species; and (iv) correlation between speciation rate and temperature across species. We estimated speciation rates using the DR statistic (Redding and Mooers 2006; Jetz et al. 2012), which has been shown to be a good approximation of recent speciation rates (Title and Rabosky 2019). We transformed these Spearman correlation coefficients to Z-scores and estimated 95$\%$ confidence intervals using Fischer transformations in the R package *DescTools* (Signorell 2021). To see if directional trends existed in the correlations, we estimated the size of the average effect for each correlation separately using random-effects models and restricted maximum-likelihood estimation in the R package *metafor* (Viechtbauer 2010). We repeated this analysis using coefficients from phylogenetic or spatial generalized least squares models which account for the non-independence of observations based on shared ancestry and spatial proximity, respectfully, and we found similar results (Supplementary Appendix S4).

### Simulation Model

We implemented four alternative models of diversification using the spatially explicit general engine for eco-evolutionary simulations, *gen3sis*, version 1.3 (Hagen, Flück et al. 2021). The simulations follow the diversification of a clade from a single ancestral species at the beginning of the Cenozoic (65 Ma; Supplementary Fig. S2). The simulations track populations as they disperse and diversify throughout 65 myr of reconstructed temperature, aridity, plate tectonic, and geomorphological changes across a gridded global landscape at 220 km $\times $ 220 km resolution (Hagen et al. 2019). Paleoenvironmental reconstructions allow us to account for dynamic changes and fluctuations of temperature over the Cenozoic, expressed via temperature, in driving variation in evolutionary speed under the different diversification models. Unlike previous simulation models testing the ESH (e.g., Hurlbert and Stegen 2014a,b), this model inherently incorporates evolutionary inertia of species environmental niches and geographical distributions in two-dimensional space, where speciation and extinction are emergent properties rather than determined probabilistically according to a birth–death model. As such, the ESH is modeled alongside other factors which may have been instrumental in shaping contemporary biodiversity patterns, such as climate change and stability (Dynesius and Jansson 2000a), or changes in the available area of suitable habitat through time (Jetz and Fine 2012). Biodiversity patterns emerge from simulated phylogenetic trees, spatial distributions of species, and trait distributions.

Following Hagen et al. (2019); Hagen, Flück et al. (2021), and Scotese et al. (2021), paleoenvironmental temperature was derived from reconstructions of Koeppen climatic belts at 5 myr intervals during the Cenozoic (Scotese et al. 2021). These belts were assigned a temperature value based on present-day averages for each belt. This temperature reconstruction approximates the change in the steepness of the latitudinal temperature gradient over deep-time (Scotese et al. 2021). To account for global fluctuations in temperature, the temperature values assigned to Koeppen climate belts were adjusted according to global average temperature curves based on oxygen isotope data (Scotese et al. 2021). Finally, to account for local variation in temperature due to topography, we applied a lapse rate to temperature values at a rate of 6.5$^{\circ}$ per 1 km of altitude based on elevation from a Paleo-digital elevation model sampled at 1 myr intervals, which includes plate tectonic movements (Scotese and Wright 2018). The final temperature values at 1 myr intervals and 1-degree spatial resolution were linearly interpolated to 170 kyr intervals and 220 km $\times $ 220 km equal area resolution using a Behrmann projection. Aridity values were taken as the sub-tropical arid Koeppen climate belt. Both temperature and aridity values were standardized between 0 and 1 in the simulation model.

At each time step ($\sim $170 kyr), each population can disperse into surrounding sites from a dispersal kernel drawn from a Weibull distribution with a fixed shape parameter ($\Psi = 2.5$) and variable scale parameter ($\Phi $; Supplementary Fig. S3). The size ($N)$ of population $i$ in site $j$ is fixed and constant at each time step and determined by (i) environmental suitability based on the species’ thermal niche, (ii) carrying capacity based on aridity, and (iii) the presence of competitors. Environmental suitability is a Gaussian function of the thermal niche, which declines with increasing distance between the temperature value in the site ($T_{j})$ and the population’s temperature optimum ($T_{i})$, following (McPeek 2007, 2008) (Supplementary Fig. S4):


(1) \begin{align*}\label{eq1} N_{ij}=K * \exp(-(T_{i} - T_{j}/\omega)^{2}), \end{align*}

where $\omega $ is a parameter that determines the strength of environmental filtering, with small values leading to a sharper decline in abundance as the species temperature niche optimum ($T_{i}$) becomes more different from the temperature of the site ($T_{j}$). $N_{ij}$ equals $K$ in the absence of competitors if population $i$ is perfectly adapted to the site. The carrying capacity for each site ($K$) is entirely independent of temperature but decreases exponentially with the aridity index in each site ($A_{j}$), according to the function:


(2) \begin{align*}\label{eq2} K=K_{c} * \exp(-1*A_{j}), \end{align*}

where $K_{c}$ is a constant (30,000) determining the maximum carrying capacity in the grid cell (Supplementary Fig. S5). The decision to limit the carrying capacity of sites by aridity was based on the assumption that water availability is one of the major limiting factors for primary productivity and population size is a function of productivity based on resource availability (Waide et al. 1999; Gillman and Wright 2006). We model a zero-sum game where sites have finite resources available, which places an ecological limit on the maximum number of individuals in a site across populations of all species present ($N_{j}$). In saturated communities ($N_{j} \ge K$), when new species colonize or become locally extinct from a site, abundances of all species are reapportioned according to the environmental suitability of each species, such that well-adapted species obtain a higher abundance than poorly adapted species, following (Hurlbert and Stegen 2014c):


(3) \begin{align*}\label{eq3} \hat{N}_{ij}=N_{ij} * \min (N_{j}, K)/N_{j}. \end{align*}

Local extinction occurs deterministically if $\hat{N}_{ij} = 0$ or stochastically as a sigmoidal function of $\hat{N}_{ij}$:


(4) \begin{align*}\label{eq4} 1/(1+\exp(-\mu_{d} * (\mu_{t} - \hat{N}_{ij}))), \end{align*}

where $\mu_{t}$ is the population size threshold below which extirpation in site $j$ becomes more likely and $\mu_{d}$ is the rate of decay of the function (Supplementary Fig. S6). $\mu_{t}$ and $\mu_{d}$ parameters were fixed across simulations. Extinction of a species occurs when it no longer occupies any sites.

Evolution of the temperature niche trait ($T_{i}$) and body size ($B_{i}$) for each independently evolving population approximates a bounded Brownian motion model of trait evolution. The traits drift randomly though time but are bound between values of 0 and 1. The value of the trait at increasing time intervals of $\delta t$ is equal to the value of the trait at time $t$ plus a value drawn from a normal distribution with a mean of 0 and standard deviation of $\sigma $. We model separate rates for temperature evolution ($\sigma_{T})$ and body size evolution ($\sigma_{B}$; Supplementary Fig. S7).

Speciation is based on an allopatric model of speciation, and populations of a species that become geographically isolated from each other diverge genetically at each time step. Under the null model (M0), where population divergence is independent of temperature and body size, the amount of genetic divergence ($g$) at each time step is drawn from a uniform distribution (0.01, 1). Diverging populations become distinct species once genetic divergence has crossed threshold $S$ (2, 10). We model the effect of migration on genetic differentiation as, if populations have secondary contact (i.e., they return to within-dispersal distance of one another), they coalesce toward genetic homogeneity at a rate of 1 per time step. We additionally model three alternative scenarios in which rates of population divergence are temperature-dependent (M1), body-size dependent (M2), or temperature and body-size dependent (M3). Under M1, the genetic divergence of populations $i$ and $k$ (g$_{i,k}$) is a function of the sum of the average temperatures ($\hat{T}$) experienced by diverging populations $i$ and $k$ across all sites within their geographic range, scaled exponentially with the parameter $\lambda $ (Supplementary Fig. S8):


(5) \begin{align*}\label{eq5} g_{i,k} = ((\hat{T}_{I} + \hat{T}_{k})/2)^{\lambda }. \end{align*}

As temperature values are standardized between 0 and 1, the maximum value of $g$ at each time step is equal to 1, and $\lambda $ determines the rate of exponential decline toward 0 as species inhabit cooler grid cells. Under M2, $g_{i,k}$ is a function of the sum of the average standardized body sizes ($\hat{B}$) of diverging populations $i$ and $k$, scaled exponentially with the parameter $\lambda $ (2, 5) (Supplementary Fig. S7):


(6) \begin{align*}\label{eq6} g_{i,k} = ((1-\hat{B}_{I} + 1-\hat{B}_{k})/2)^{\lambda}. \end{align*}

Here, genetic divergence exponentially approaches a value of 1 as body size decreases, at a rate of $\lambda $. Finally, under M3, $g_{i,k}$ is a function of the average of $1-\hat{B}$ and $\hat{T}$ (hereafter $\hat{B}\hat{T}$), scaled exponentially with the parameter *$\lambda $*, such that genetic divergence is faster in small-bodied populations in warmer regions and decreases exponentially as species increase in body size and occupy cooler sites (Supplementary Fig. S7):


(7) \begin{align*}\label{eq7} g_{i,k} = ((\hat{B}\hat{T}_{I} + \hat{B}\hat{T}_{k})/2)^{\lambda }. \end{align*}

We ran the simulation model 500 times under each of the four scenarios, varying six key parameters: the divergence threshold [$S$, parameter range $=$ (2, 10)], the rate scaling factor for the rate of population divergence under M1–M3 [$\lambda $, (2, 5)], the strength of environmental filtering for the temperature niche trait [$\omega $, (0.01, 0.035)], the rate of body-size evolution under Brownian motion [$\sigma_{B}$, (0.001, 0.02)], the rate of temperature niche evolution [$\sigma_{T}$, (0.001, 0.015)], and the dispersal kernel [$\Phi $, (330, 880)]. These parameter ranges were determined via preliminary examination of the simulation model to broadly cover a range of conditions while consistently generating clades of comparable size to the empirical data ($\sim $20–6000 species; Supplementary Fig. S9). The model is computationally intensive, which restricts the number of replicates possible. To accommodate this limitation, we used a quasi-random sampling technique to select parameter combinations that evenly cover the six-dimensional parameter space (approximating a uniform distribution for each parameter) using Sobol sequences (Burhenne et al. 2011) and assessed the subsequent parameter sensitivity. It has been shown that strategies that sample parameters broadly and evenly across multidimensional parameter space are efficient for exploring stochastic simulation models (Prowse et al. 2016).

### Model Validation and Sensitivity Analysis

We estimated the same 54 biodiversity summary statistics on the simulated data as calculated for the empirical data (Supplementary Table S1). We then assessed the validity of the model by comparing the univariate distributions of the simulated and empirical summary statistics (Supplementary Fig. S10), as well as the overlap in multivariate space using principal component analysis (PCA; Supplementary Fig. S11). We investigated model behavior and the relationships between model parameters and summary statistics using global sensitivity analysis, following the procedure of (Prowse et al. 2016) (Supplementary Figs. S12–S14), as well as visualizing the relationships between model parameters and summary statistics in multivariate space using PCA (Supplementary Figs. S11 and S15). We removed seven summary statistics which were not well captured by the simulation model, as well as 23 variables that were highly collinear (Pearson’s $r > 0.90$; Supplementary Fig. S16), from the dataset, leading to 26 summary statistics for downstream analyses. For a detailed description of the simulation model, experimental design, model validation, and sensitivity analysis, please see the Supplementary Methods in Appendix S3.

### Model Discrimination

After characterizing how biodiversity patterns are generated by the model parameters and checking the validity of the model in capturing realistic biodiversity patterns, we asked whether biodiversity patterns differ between the population divergence models. We subset the complete simulation dataset to include only those simulations whose parameters led to complete simulations under all four models of population divergence ($n = 1384$) to ensure equal sample sizes between model classes. We used supervised machine learning model classification tools with 10-fold cross-validation repeated 10 times on a two-thirds training subset of the simulated data ($n = 917$). We then estimated reclassification accuracy based on model predictions on the withheld one-third test dataset ($n = 467$). To investigate the robustness of the results to different machine learning algorithms, we used the R package *Caret* to repeat this procedure for seven different classification algorithms: linear discriminant analysis, three decision tree algorithms (recursive partitioning and regression trees, random forest, and a gradient-boosting machine algorithm), naïve Bayes, support-vector machines, and neural networks. We assessed model reclassification accuracy (Supplementary Table S2) and ranked the different classification algorithms using global accuracy and Cohen’s $\kappa $ metrics (Supplementary Fig. S17). We investigated which biodiversity summary statistics were most important in separating the models of population divergence by looking at the relative contribution of each summary statistic using variable importance factors.

### Model Selection

As a final step, we fitted the machine learning classification models to the empirical data, estimated the relative support for each population divergence model using all seven machine learning algorithms, and weighted these estimates using Cohen’s $\kappa $ to obtain model-averaged support for each population divergence model in each clade. To interrogate the role of clade age in influencing the model selection results, we repeated the model selection procedure on all time-slice clades. Furthermore, although the range of clade sizes was similar between simulated and empirical datasets (Supplementary Fig. S9), the distribution was different, with empirical data being far more right skewed toward smaller sizes (simulated skewness $= 0.23$, empirical skewness $= 4.45$; Supplementary Fig. S9). To investigate whether this difference in clade size distributions influenced the results, we repeated the model selection procedure by training the machine learning models on a subset of the simulated data that produced clades with fewer than 1000 species and repeated the predictions. Finally, to compare the impact of the method of phylogenetic metric calculations, we repeated the model selection procedure using phylogenetic metrics calculated on the MCC tree, or the mean values from across a sample of 50 trees from the posterior distribution. In all these analyses, the results were qualitatively similar and are presented in the Supplementary Appendix (Supplementary Table S3 and Fig. S18).

## Results

### Empirical Biodiversity Patterns

**Figure 3.**
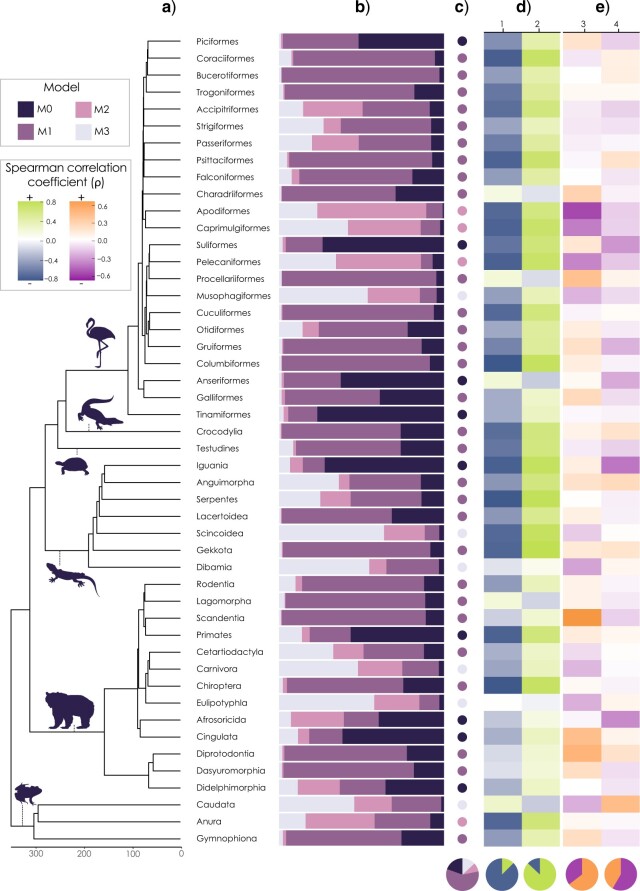
Empirical support based on 26 summary statistics for population divergence models (M0–M3) and the distribution of four key summary statistics in tetrapod orders. a) Phylogenetic relationships of 48 orders of birds, mammals, amphibians, and reptiles (squamate reptiles divided into six infraorders) with b) $\kappa $-weighted averaged posterior support from seven different model classification algorithms for each population divergence model and c) the best supported population divergence model shown in colored circles. Circle graph beneath this column shows the proportion of clades with support for each model. d) Spearman correlation coefficients ($\rho )$ between species richness and (1) latitude and (2) temperature, measured at 220 km $\times $ 220 km grid cells. Circle graphs show the proportion of positive and negative correlations. e) Spearman correlation coefficients ($\rho )$ between speciation rate (measured using the DR statistic) and (3) body size and (4) and temperature measured at species-level. Circle graphs show the proportion of positive and negative correlations.

Meta-analysis of Spearman correlation coefficients between temperature and species richness and between latitude and species richness, as measured across 220 km $\times $ 220 km grid cells, showed that general effects were directional, with temperature showing a positive net effect on species richness ($\beta = 0.169 \pm 0.046, Z = 3.688, P = 0.0002$) and latitude showing a negative net effect on species richness ($\beta = -0.230 \pm 0.0581, Z = -3.951, P < 0.001$; Fig. 3d). Meta-analysis of trends in the relationship between species-specific estimates of speciation rates, and body size and between speciation rates (DR) and temperature, showed that, unlike the spatial correlations, these species-level correlations did not show any directional trends among tetrapods (temperature $\sim $ DR, $\beta = -0.009 \pm 0.008, Z = -1.089, P = 0.28$; body size $\sim $ DR, $\beta = 0.0003 \pm 0.0003, Z = 0.972, P = 0.331$). These results were also supported when considering the non-independence of observations using spatial or phylogenetic generalized least squares models (Supplementary Appendix S4).

### Model Discrimination

**Figure 4.**
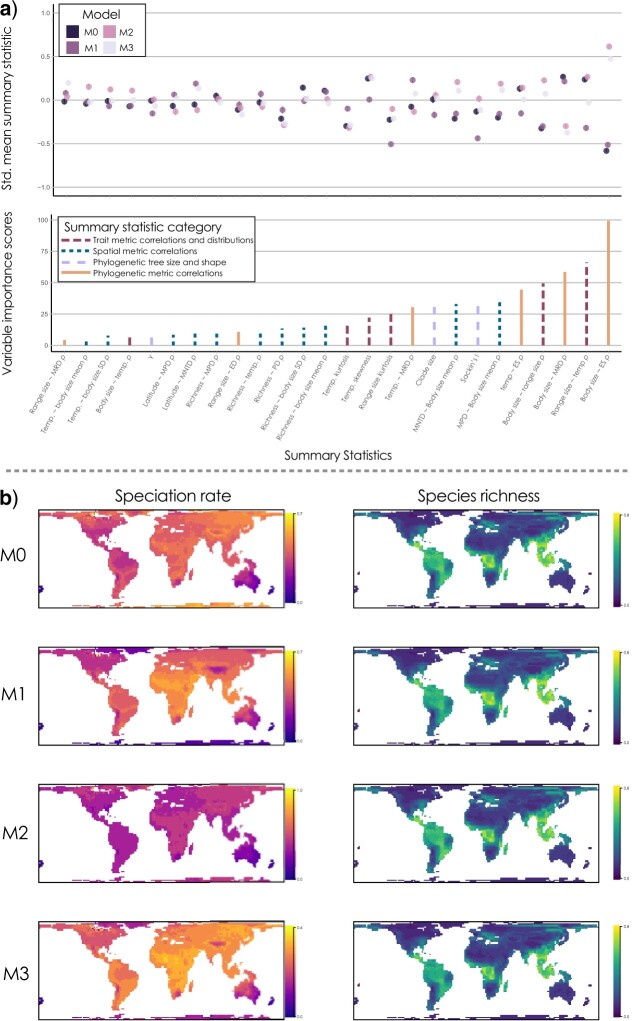
a) Scaled variable importance scores for 26 summary statistics based on model averaged variable importance factors from seven machine learning algorithms (bottom panel). Distribution of mean $\pm $ standard error of 26 summary statistics across four population divergence models (top panel). Different classes of summary statistics vary in their relative importance, for example, summary statistics based phylogenetic metrics ES or MRD which relate to speciation rates (left hand column, b) tend to differ strongly between models and be highly informative, whereas summary statistics based on spatial correlations, particularly species richness (right hand column, b), tend to be uninformative. Phylogenetic metric correlations are correlation coefficients between phylogenetic tip metrics (MRD, ES, ED, DR) and species-level traits (geographic range size, mean temperature, body size). Spatial correlations are correlation coefficients between grid-cell level properties of assemblages (species richness, MPD, MNTD, PD, mean body size, standard deviation of body size) and environmental properties (latitude or temperature). Trait correlations and distributions relate to characteristics of species-level traits (body size, geographic range size, mean temperature). Phylogenetic tree size and shape relates to clade size and the distribution of node heights ($\gamma )$ or imbalance (Sackin’s I). b) The distribution of species-specific speciation rates (grid cell averaged DR across species, left hand column) and species richness (right hand column) across four population divergence models incorporating historical paleoenvironmental changes (M0–M3). Grid cell averaged values from each simulation were fist standardized to be between 0 and 1 before an overall mean was taken for all complete simulations. ED $=$ evolutionary distinctiveness measured using the fair proportion measure; ES $=$ equal splits measure of ED; DR $=$ diversification rates measured as the inverse of ES; MRD $=$ mean root distance, MNTD $=$ mean nearest taxon distance; MPD $=$ mean pairwise distance; PD $=$ phylogenetic diversity.

Biodiversity patterns in the simulations varied between population divergence models, leading to high discrimination ability of machine learning classification algorithms. Using 10-fold cross-validation repeated 10 times with 7 different model classification algorithms, we found a high proportion of correctly identified population divergence models, with classification considered in moderate (McHugh 2012) to substantial (Cohen 1960) agreement based on Cohen’s $\kappa $ (global accuracy [0.66, 0.78]; Cohen’s $\kappa $ [0.54, 0.71]). We found that different model classification algorithms varied in their performance, with models allowing complex relationships between variables (e.g., neural networks, gradient boosting models, and support vector machines) having higher global accuracy (Supplementary Fig. S17). We also found that for all algorithms, there was variation in prediction accuracy across model classes (Supplementary Table S2), with M3, which combines the temperature-dependent divergence of M1 and body-size-dependent divergence of M2, having lower rates of true positives (sensitivity) and true negatives (specificity; Supplementary Table S2). We found that summary statistics contributing most to the discrimination ability, based on variable importance factors from each classification algorithm weighted by the $\kappa $ value from each algorithm, represented several different categories of summary statistic (Fig. 4a). For example, the two summary statistics with the highest variable importance scores were a correlation between body size and the equal splits measure of evolutionary distinctiveness (a proxy for speciation rate), and a correlation between geographic range size and temperature, which represent phylogenetic metric correlations and trait metric correlations, respectively. In contrast, some biodiversity summary statistics only weakly contribute to model discrimination, including a correlation coefficient between species richness and temperature. A negative correlation between absolute latitude and species richness and a positive relationship between temperature and species richness emerged with all models (Fig. 4b).

### Model Selection

Using seven model classification algorithms, we estimated the population divergence model of best fit (Fig. 3c) as well as the proportional support for each model (Fig. 3b) across 48 tetrapod orders. We found that under all seven algorithms, a population divergence model that included temperature dependency (M1) was the best fitting model in the largest number of clades. This varied between 16 clades with the neural network algorithm to 38 clades with the naïve Bayes algorithm. To incorporate uncertainty between classification model algorithms, we took a model averaged estimate of model support, by weighing model support by $\kappa $ (Fig. 2b,c; Supplementary Table S4). We found M1 was best supported in the largest number of clades (29 of 48), followed by the null model (M0; 9 clades), the temperature- and body-size-dependent model (M3; 6 clades), and the body-size-dependent model (M2; 4 clades). Therefore, 35 clades (74.4$\%$) had the strongest support for a population divergence model that included temperature dependency (M1 and M3), compared with no dependency (M0) or body-size dependency only (M2). This general support for M1 in the data was stronger when considering time-slice clades, or when training the model only using low diversity simulations (Supplementary Table S2). Estimating the relative proportion of model support across classes allowed us to look at the uncertainty implicit in model selection and we found that the strength of support for each model was variable. In some clades, support was predominantly attributed to a single model (e.g.,, M1 in the hornbills and allies, Bucerotiformes; Fig. 3b), whereas in other clades, support was more evenly distributed across population-divergence models (e.g., support was split between M2 and M3 in the nightjars and allies, Caprimulgiformes; Fig. 3b).

## Discussion

Temperature is thought to play an integral role in shaping rates of speciation by driving rates of molecular evolution and subsequent speciation in diverging populations (Rohde 1992). Despite a strong theoretical underpinning (Allen et al. 2002; Brown 2004), a positive link between environmental energy, in the form of temperature (or otherwise), and rates of diversification has rarely been observed in large comparative datasets (Jetz et al. 2012; Rabosky et al. 2018). As such, current evidence favors alternative mechanisms in shaping biodiversity patterns, such as evolutionary time (Marin and Hedges 2016; Miller et al. 2018), ecological limits (Rabosky and Hurlbert 2015) (but see Harmon and Harrison 2015), or an effect of geographic area over time (Fine and Ree 2006; Jetz and Fine 2012). Yet, tests of the ESH typically treat temperature as a static feature of the present-day, rather than considering how dynamic changes in temperature over deep-time scales may influence diversification dynamics. Furthermore, highly dimensional biodiversity patterns, integrating traits, spatial distributions, and phylogeny are rarely used to draw inferences on macroevolutionary patterns. Contrary to findings from earlier studies, by combining spatially explicit simulation models that incorporate historical fluctuations in temperature and simulation-based inference tools, we found common support for a diversification model including temperature-dependent divergence across tetrapods. This support was estimated from a suite of commonly used biodiversity summary statistics, reflecting spatial, trait, and phylogenetic patterns, highlighting that a diverse array of summary statistics is needed to diagnose models of population divergence from commonly used biodiversity data.

A general effect of temperature (M1) was supported across ecologically and geographically diverse tetrapod clades with different thermoregulatory modes (ectothermy and endothermy), whereas only weak support for the role of body size was found (M2 and M3). The ESH, as originally put forward by Rohde, argued that evolutionary rates are dependent on both temperature and life history (specifically generation time) (Rohde 1992). There is some empirical evidence for a relationship between body size (and associated life history traits) and substitution rates in vertebrates (Martin and Palumbi 1993), and we found support for a body-size-dependent model of population divergence (M2 and M3) in several large radiations, including skinks and allies (Scincoidea) and frogs (Anura; Fig. 3c). However, more generally, these models (M2 and M3) received only low support in most clades (Fig. 3). This is partly because correlations between body size and the equal splits measure of evolutionary distinctiveness (Redding and Mooers 2006; Jetz et al. 2012), the most important summary statistic for model discrimination (Fig. 4), had higher values in M2 and M3 than the empirical data, which was more closely matching M0 and M1. Instead, our results show the greatest support for a predominant role of temperature in shaping rates of divergence (M1). This was, in addition to the ES-richness correlation, also strongly determined by a more negative correlation between geographic range size and temperature in both empirical and M1 data—the second most important summary statistic (Fig. 4). This is expected if speciation is temperature-dependent because, if allopatric speciation is a process which divides species geographic ranges, and speciation happens faster in warm places, then warm places should have more species with overall smaller geographic ranges. Therefore, our results suggest that temperature-dependent speciation could contribute to Rapoport’s rule—the observation that geographic ranges become smaller toward the tropics (Stevens 1989)—and that this phenomenon is not inconsistent with the ESH (Rohde 1992, 1996).

The estimated support for a temperature-dependent model of population divergence provides support for the ESH; however, it remains an open question exactly how temperature shapes rates of divergence in real clades. As originally described by Rohde (1992), the ESH makes predictions based on largely on variation in rates of molecular evolution with temperature. Empirical support for greater UV-damage-driven molecular evolution is weak, as it should also apply to high-elevation lineages, which have showed the opposite pattern of lower rates (Wright et al. 2010). Although the basal metabolic-pathway explanation makes separate predictions for ectotherms and endotherms (which we do not see in our results), as endotherms maintain a constant body temperature across environmental temperature gradients (Gillman et al. 2009). We therefore find these explanations unlikely. On the other hand, variation in annual metabolic rates, which are expected to be lower in species occupying more seasonal climates which undergo hibernation or torpor during colder months (Gillman and Wright 2013), remains a plausible explanation.

It is also possible that population divergence in real clades may be accelerated by an unmeasured factor that is correlated with temperature but uncorrelated (or weakly correlated) with body size. For example, some hypotheses and empirical data suggest population sizes are expected to be smaller in warmer regions because increased productivity drives negative density-dependent population dynamics from greater competition, predation, or trophic diversity (Paine 1966; Janzen 1970; Connell and Orias 1964), leading to faster rates of molecular evolution (Kimura 1983; Ohta 1992; Woolfit 2009). Alternatively, greater productivity could increase population sizes (Storch et al. 2018), leading to more genetic diversity and standing variation for selection to act upon (Fine 2015). Another theory, based on the red queen hypothesis (Van Valen 1973), is that lineages in high-energy environments have higher rates of divergence as a result of strong divergent selection from interactions with different species (Gillman et al. 2009; Schemske et al. 2009). Taken together, our results suggest an integral role of temperature in driving rates of population divergence and generating biodiversity patterns. However, the exact mechanism differs from that predicted by the original ESH (Rohde 1992), with rates dependent on body size as a proxy for life history being less supported. Further work disentangling the drivers of faster rates of divergence in warm areas, whether from population size effects related to productivity or biotic interactions, is a key next step.

We found overall support for temperature-dependent rates of population divergence in terrestrial vertebrates, despite observing only weak support for that mechanism from individual summary statistics. Meta-analyses of correlation coefficients in tetrapods showed that, despite an overall significant and positive effect of temperature on species richness which is consistent with findings from previous studies (Currie 1991; Belmaker and Jetz 2011; Skeels et al. 2019), there was no significant effect of temperature on speciation rates (Fig. 3). In fact, using PGLS, only 10 clades showed a significant positive correlation between temperature and species-specific speciation rates (DR statistic [Jetz et al. 2012]), compared with 13 clades showing a significant negative correlation—with rates being higher in lineages occupying colder regions (Supplementary Appendix S4). This result matches several recent studies in which higher diversification rates were found in higher-latitude regions (with lower temperatures) (Weir and Schluter 2008b; Rabosky et al. 2018). This may reflect a geographic bias in how species are taxonomically described (Freeman and Pennell 2021), an artifact of studying rate variation over short timescales (Harmon et al. 2021), or it may reflect genuinely different mechanisms operating across latitudes (Cutter and Gray 2016). Our simulations help to elucidate this point and can also explain why we see support for temperature dependency, despite weak or inconsistent individual patterns. Holding the rates of population divergence constant, speciation in the null model (M0) is only a function of the rate of population isolation, and here we see the highest rates at higher latitudes. When introducing a temperature-dependent rate of population divergence (M1), we see the distribution of high speciation rates becoming more equatorial, with some of the highest values occurring in the deserts and grasslands of the Afrotropics where richness is often low (Fig. 4b). Yet even under M1, correlations between speciation rates and temperature were highly variable and often negative. This tells us that rates of population isolation are greater in colder regions, but this pattern can be counter-balanced by rapid population divergence in warmer regions, highlighting a potentially very important effect of climatic stability on the formation of biodiversity (Pianka 1966a; Dynesius and Jansson 2000b). The absence of ice-cover in our model inputs may also affect this result, as the only constraint on dispersal in polar regions is the temperature niche of species. These processes also lead to incongruence between regions of high species richness, high temperatures, and high speciation rates (Fig. 4b). Therefore, support for the ESH drawn from the spatial distribution of speciation rates may be at risk of misinterpreting the strong signal of recent population fragmentation as an absence of evidence of temperature-driven population divergence. Our results suggest that the spatial distribution of speciation rates should be interpreted cautiously, ideally with the simultaneous assessment of multiple biodiversity patterns, before drawing inferences.

Predictive models should match different patterns simultaneously because evolutionary processes have downstream impacts on a whole suite of biodiversity patterns (Gallagher et al. 2021; Hagen, Flück et al. 2021). In this study, we used an uncorrelated subset of 26 out of 54 different summary statistics to perform inference, which covered a vast array of biodiversity patterns. These included some of the most well-known and ubiquitous macroecological patterns, including the relationship between latitude and geographic range size (Rapoport’s rule [Stevens 1989]), between body size and temperature (Bergmann’s rule [Bergmann 1847]), and between latitude and species richness (the latitudinal diversity gradient [Hillebrand 2004]). These kinds of macroecological patterns are not always considered when testing macroevolutionary theories such as the ESH. However, any evolutionary process that generates biodiversity and operates over deep time should leave detectable signatures not only in the shape of phylogenetic branching patterns and their correlates, but also in spatial diversity patterns (McGill et al. 2019).

We found that most summary statistics showed very high congruence between simulated and empirical datasets, supporting the validity of the model in generating realistic patterns. We also found that many broadscale macroecological patterns differed between population divergence models, for example, the correlation between geographic range size and temperature was considered the second most important explanatory summary statistic (Fig. 4a), and the combination of the most informative summary statistics considered different dimensions of biodiversity (Fig. 4a). However, some summary statistics, including correlations between species richness and latitude or temperature, showed little variation between population divergence models (Figs. 2d and 4b). The ESH was originally formulated as an explanation for the latitudinal diversity gradient based on the premise that other explanations, such as environmental stability (Pianka 1966b), biotic interactions (Dobzhansky 1950), geographic area (Rosenzweig 1995), and evolutionary time (Fischer 1960), have weak or partial explanatory power (Rohde 1992). Here, we argue that although there is strong support for the role of temperature-dependent evolutionary rates in the formation of multiple biodiversity patterns, the ESH is not necessarily the primary cause of the latitudinal diversity gradient, as this pattern is indistinguishable between the null and alternative models (Fig. 4). However, we note that local abundances in our model were determined by three factors, the match between environmental temperature and the thermal niche of species, the aridity of the site, and the presence of other species. Hence, the potential species richness of a site at equilibrium would be environmentally determined, which explain similar patterns of species richness between models, despite underlying differences in macroevolutionary rates. This fits with the understanding that equilibrium effects can mask historical dynamics for particular metrics, such as species richness; however, notable differences between other summary statistics suggest that historical effects can be detected with a multivariate metric approach.

### Caveats and Future Directions

The common support for a temperature-dependent model was robust to several different analysis strategies, including taxonomic and clade size sampling strategies (Supplementary Appendix S4); however, there are features of the simulation models and empirical data that may still introduce biases to the results. The simulations varied in the models of population divergence yet had the same functions for dispersal, trait evolution, and ecological interactions. Where possible, we selected functions that have been used successfully in the literature before. For example, models including environmentally determined carrying capacities yield a consistently better fit to empirical data across different kinds of simulation models (Hurlbert and Stegen 2014b; Hagen, Flück et al. 2021). Yet some models may incompletely represent real processes. For example, a Brownian Motion model of trait evolution does not always produce the right skew in body size distributions of real clades (Kozłowski and Gawelczyk 2002), and summary statistics of the frequency distribution of body size in our study were some that showed the least congruence with empirical data. The field of spatially explicit simulation modeling is emerging and exploration of different kinds of ecological models is still in its infancy (Pontarp et al. 2019). As such, some modeling decisions were not exhaustively explored in this study, such as the starting time and distribution of the initial species in the simulations, as well as alternative paleoenvironmental reconstructions.

One specific process that may help to further elucidate the mechanisms underlying observed support for the temperature-dependent model is incorporating an effect of population size on population divergence directly. In this study, we model population size based on the thermal niche requirements of the species and limiting water availability, but for simplicity, we do not model an interaction between population size and population divergence. Population size may influence evolutionary speed via its effect on the rate in which different kinds of mutations go to fixation (Lanfear et al. 2014; Hua and Bromham 2017), with the expectation that beneficial mutations are fixed at a higher rate in larger populations and slightly-deleterious mutations are fixed at a higher rate in small populations, but the rate of substitution for effectively neutral mutations is independent of population size (Lanfear et al. 2014). This means that if most mutations are slightly deleterious the average rate of molecular evolution should decrease with increasing population size (Lanfear et al. 2014), such that smaller populations might diverge more rapidly from one another, leading to faster rates of speciation. Alternatively, the integrated ESH (Gillman and Wright 2013) predicts that a greater efficiency of fixing beneficial mutations in larger populations may lead to greater adaptive potential, leading to local adaptation and ecological divergence between populations, driving more rapid speciation. A subsequent prediction of the integrated ESH is that aridity not only effects population size, but also might indirectly drive variation in evolutionary speed, and this is supported in some taxa (Goldie et al. 2010). These two models are amenable to testing using the simulation-based approach taken here and this possible future direction may help clarify the most likely effect of population size on evolutionary speed.

Another potential source of bias may arise from the analysis of congruence between simulated and empirical data. Simulated data represented a perfectly known history of a clade, whereas empirical data are incompletely sampled and contains both measurement error in spatial and trait data as well as potential biases in phylogenetic reconstructions. If biases present in empirical sampling are biased with respect to traits, such as body size, then this may impact subsequent correlations with other features such as diversification rates, particularly if these features are intercorrelated (e.g., smaller body sized organisms diversify faster). We used body size as a trait representing life history as this trait is very well sampled across tetrapods (Etard et al. 2020), but sampling for this trait as well as molecular data used in phylogenetic inference are more sparse in herptile clades than birds and mammals (Jetz et al. 2012; Tonini et al. 2016; Jetz and Pyron 2018; Upham et al. 2019; Etard et al. 2020). Herptile clades have fundamentally different predictions under the metabolic-theory interpretation of the ESH based on their thermal regulatory mode, and potential biases here might have important consequences. Nonetheless, sampling of body size in herptile families is greater than 80$\%$ (Etard et al. 2020) and we suspect the main relationships should be robust to this taxonomic bias. Sampling bias across taxa highlights the value of field-based and data collection studies that support large comparative studies such as this one.

Finally, we acknowledge that our approach to assessing the ESH hypothesis prevented us from and explicit separation of, and comparison with, key alternative hypotheses such as the past dynamics in the available area of suitable habitat and productivity (Jetz and Fine 2012) and in climate (Dynesius and Jansson 2000a). These hypotheses inherently include effects of temperature and redeveloping the simulation methodology to cleanly delineate these signals was outside the scope of this work which had a focus on evolutionary speed. Studies which include counter-factual case-studies provide a potentially valuable method of separating confounding effects. For example, some previous simulation studies modified historical landscapes to remove the effects of mountain building (Rangel et al. 2018), or aridification (Hagen, Skeels et al. 2021), to explore how diversification may have proceeded had these Earth history events never occurred. In this vein, we propose that removing historical climate and landscape changes or modifying their velocities could offer a way to separate the effects. We highlight this as a promising area for future work.

## Conclusion

By explicitly considering evolutionary and ecological mechanisms alongside dynamic changes in plate tectonics and temperature over the Cenozoic using a simulation-based inference approach, we found strong support that temperature-dependent population divergence shapes speciation rates and broad-scale biodiversity patterns across tetrapods. Counter-intuitively, we show that a positive relationship between temperature and species richness does not provide sufficient evidence that temperature plays a generative role in lineage diversification, whereas a negative relationship between temperature and speciation rate does not provide sufficient evidence to negate a generative role of temperature in lineage diversification. Instead, model support was derived from numerous summary statistics, highlighting that multiple lines of evidence should be combined before precluding specific mechanisms. This has important implications; given that most real clades show weak relationships between temperature and diversification rates (Fig. 3e), dismissals in previous studies of the role of temperature in shaping evolutionary speed must be reassessed. Simulation-based approaches, such as the one used in this study, allow us to compare complex and spatially-dynamic models of evolution and place uncertainty intervals on different evolutionary mechanisms. We can now ask, in light of multidimensional biodiversity patterns, which evolutionary mechanisms appear more probable. We hope that future studies might extend this hypothesis testing framework using spatial simulation models to test more hypotheses for the formation of global biodiversity patterns, enabling us to move toward a more comprehensive assessment of the processes generating the extraordinary diversity of life today.

## Supplementary Material

Data available from the Dryad Digital Repository: https://doi.org/10.5061/dryad.cnp5hqc71

## Data and Code Availability Statement

No new data were generated for this study and all data used were from publicly available data sources. All simulations and analyses were performed in R, version 4.0.3 (R Core Team 2020). All R scripts to replicate the simulations and analyses from the empirical data can be found on GitHub (www.github.com/alexskeels/gen3sis_evolutionary_speed) and are also available from the Dryad Digital Repository: https://doi.org/10.5061/dryad.cnp5hqc71.
